# Comparative thromboembolic risk of romiplostim versus eltrombopag in immune thrombocytopenia: a propensity score-matched cohort study

**DOI:** 10.1007/s00277-026-07160-4

**Published:** 2026-07-03

**Authors:** Anand Shah, Rajvi Gor, Joshua Kra

**Affiliations:** 1 Department of Medicine, Rutgers New Jersey Medical School, Newark, NJ USA; 2 Department of Hematology/Oncology, Moffitt Cancer Centre, University of South Florida, Tampa, FL USA; 3 Rutgers Cancer Institute of New Jersey, Newark, NJ USA

**Keywords:** Immune thrombocytopenia, Romiplostim, Eltrombopag, Thromboembolism, Thrombopoietin receptor agonists, Propensity score matching

## Abstract

Immune thrombocytopenia (ITP) is associated with both bleeding and thrombotic complications. Thrombopoietin receptor agonists (TPO-RAs), particularly romiplostim and eltrombopag, are widely used as second-line therapy, yet comparative data on thromboembolic risk between these agents remain limited. We conducted a retrospective cohort study using the TriNetX federated research network, including adults with ITP who initiated romiplostim or eltrombopag between January 2010 and December 2025. ITP was defined using ICD-10 code D69.3. Patients with prior TPO-RA exposure, alternative thrombocytopenia etiologies, or thrombotic events within 1 year before treatment initiation were excluded. One-to-one propensity score matching was performed using demographics, comorbidities, prior therapies, concomitant medications, and laboratory variables. After matching, 3,055 patients remained in each group. Romiplostim was associated with a higher cumulative risk of any thromboembolism than eltrombopag at 30 days (7.2% vs. 5.7%; RR 1.27, 95% CI 1.05–1.54), 90 days (10.7% vs. 7.9%; RR 1.36, 95% CI 1.16–1.59), 1 year (14.9% vs. 12.2%; RR 1.22, 95% CI 1.07–1.39), and full follow-up (22.5% vs. 19.2%; RR 1.17, 95% CI 1.06–1.29). Portal vein thrombosis was more frequent with romiplostim at 1 year, although absolute event rates were low (RR 2.32, 95% CI 1.36–3.96). Mean platelet counts were similar at most time points. These findings suggest that romiplostim may be associated with higher cumulative thromboembolic risk than eltrombopag despite comparable platelet counts. Given the retrospective design and potential confounding, these results should be interpreted as hypothesis-generating.

**Supplementary Information:**

The online version contains supplementary material available at https://doi.org/10.1007/s00277-026-07160-4.

##  Introduction

Immune thrombocytopenia (ITP) is an acquired autoimmune disorder characterized by isolated thrombocytopenia resulting from increased platelet destruction and impaired platelet production [1]. Although bleeding is its major clinical complication, ITP is also associated with an increased risk of venous and arterial thromboembolism compared with the general population [1, 2]. Proposed mechanisms include elevated factor VIII and von Willebrand factor levels, preactivated platelets, and enhanced thrombin generation potential [2].

Approximately 20% of patients achieve long-term remission with corticosteroids, the standard first-line therapy, while the majority require second-line therapy [3]. Thrombopoietin receptor agonists (TPO-RAs), particularly romiplostim and eltrombopag, are among the most commonly used second-line agents, and current guidelines recommend them over rituximab or splenectomy in appropriate patients through shared decision-making [4, 5]. In randomized controlled trials, both agents have demonstrated high initial and durable platelet response rates [6, 7]. However, thromboembolism remains one of the principal safety concerns associated with TPO-RA therapy. Extension studies have reported thromboembolic events in approximately 6% of treated patients over a median follow-up of 2 years, and a recent meta-analysis suggested that thrombotic risk may increase with longer treatment duration [8–10].

Despite more than a decade of clinical use, no randomized controlled trial has directly compared romiplostim and eltrombopag for efficacy or safety outcomes [1, 11]. As a result, treatment selection is often guided by route of administration and patient preference. Existing indirect-comparison meta-analyses have suggested broadly similar efficacy and overall safety profiles but were not powered to detect differences in relatively uncommon thrombotic events [11, 12]. Pharmacovigilance data suggest the thrombotic risk may differ between agents. A disproportionality analysis of the WHO VigiBase found higher thrombosis reporting with eltrombopag than romiplostim (adjusted ROR 1.72; 95% CI 1.47–2.02), and a subsequent FAERS analysis identified different thromboembolic patterns for each drug, with romiplostim associated more strongly to arterial embolism and eltrombopag to renal embolism. These studies, however, cannot adjust for confounders or account for reporting biases, limiting their ability to establish causation [13, 14].

Real-world comparative data specifically evaluating thrombotic outcomes between romiplostim and eltrombopag in ITP remain limited [15, 16]. We therefore conducted a propensity score-matched cohort study using the TriNetX federated research network to compare thromboembolic outcomes and platelet response between these agents in adults with ITP.

##  Methods

We conducted a retrospective cohort study using the TriNetX federated research network, a multi-institutional de-identified electronic health record database encompassing 120 healthcare organizations in the United States [17]. Adults (≥ 18 years) diagnosed with ITP between January 2010 and December 2025 who initiated romiplostim or eltrombopag were identified. ITP was defined using TriNetX diagnostic coding for ITP (ICD-10 code D69.3). The index date was defined as the date of first recorded romiplostim or eltrombopag prescription/order. Patients with prior TPO-RA exposure were excluded to approximate a new-user design. Additional exclusions included diagnoses suggesting alternative thrombocytopenia etiologies, including coded secondary thrombocytopenia, myelodysplastic syndrome, heparin-induced thrombocytopenia, hemolytic uremic syndrome, and thrombotic microangiopathy, as well as any thrombotic event within 1 year before the index. SLE diagnosis alone was not used as an exclusion criterion and was instead included as a baseline covariate in the propensity score model. The ICD-10 diagnosis codes used for cohort definition, exclusions, outcomes, and diagnosis covariates are listed in Supplementary Table S1.

Patients were analyzed according to the initially prescribed TPO-RA using an as-initiated design. Follow-up was anchored to the index agent because treatment discontinuation, switching, adherence, and dose modification could not be reliably characterized within the TriNetX platform. One-to-one nearest-neighbour propensity score matching without replacement was performed using a caliper 0.1 on the logit scale. Baseline covariates measured within 1 year before the index date included age, sex, race/ethnicity, hypertension, type 2 diabetes mellitus, obesity, chronic kidney disease, neoplasms, atrial fibrillation/flutter, systemic lupus erythematosus, tobacco use, prior splenectomy, corticosteroids, intravenous immunoglobulin, rituximab, apixaban, rivaroxaban, warfarin, aspirin, clopidogrel, atorvastatin, contraceptives, platelet count, hemoglobin, alanine aminotransferase, and aspartate aminotransferase. Laboratory variables were included based on data available within TriNetX, with missing data handled according to platform defaults. Covariate balance was assessed using standardized mean differences, with values < 0.10 considered acceptable.

The primary outcome was any thromboembolism, defined as a composite of all venous and arterial thromboembolic outcomes included in the study. Venous thromboembolism included pulmonary embolism, deep vein thrombosis, portal vein thrombosis, and other venous embolism and thrombosis. Arterial thromboembolism included acute myocardial infarction, cerebral infarction, and arterial embolism and thrombosis. Pulmonary embolism, deep vein thrombosis, acute myocardial infarction, cerebral infarction, and portal vein thrombosis were also reported individually as secondary outcomes. Outcomes were assessed cumulatively from the index date through 30 days, 90 days, 1 year, and full follow-up. The analysis was designed to compare cumulative risks at prespecified time points rather than to estimate time-to-event hazards; therefore, Kaplan-Meier curves and Cox proportional hazards models were not performed. Platelet response was evaluated using mean platelet count at the same time windows as a laboratory marker to contextualize thrombotic outcomes. Binary outcomes were reported as risk ratios with 95% confidence intervals, and continuous outcomes were compared using independent-samples t tests. A two-sided p value < 0.05 was considered statistically significant. The study used de-identified data and was exempt from institutional review board review.

##  Results

**Fig. 1:**
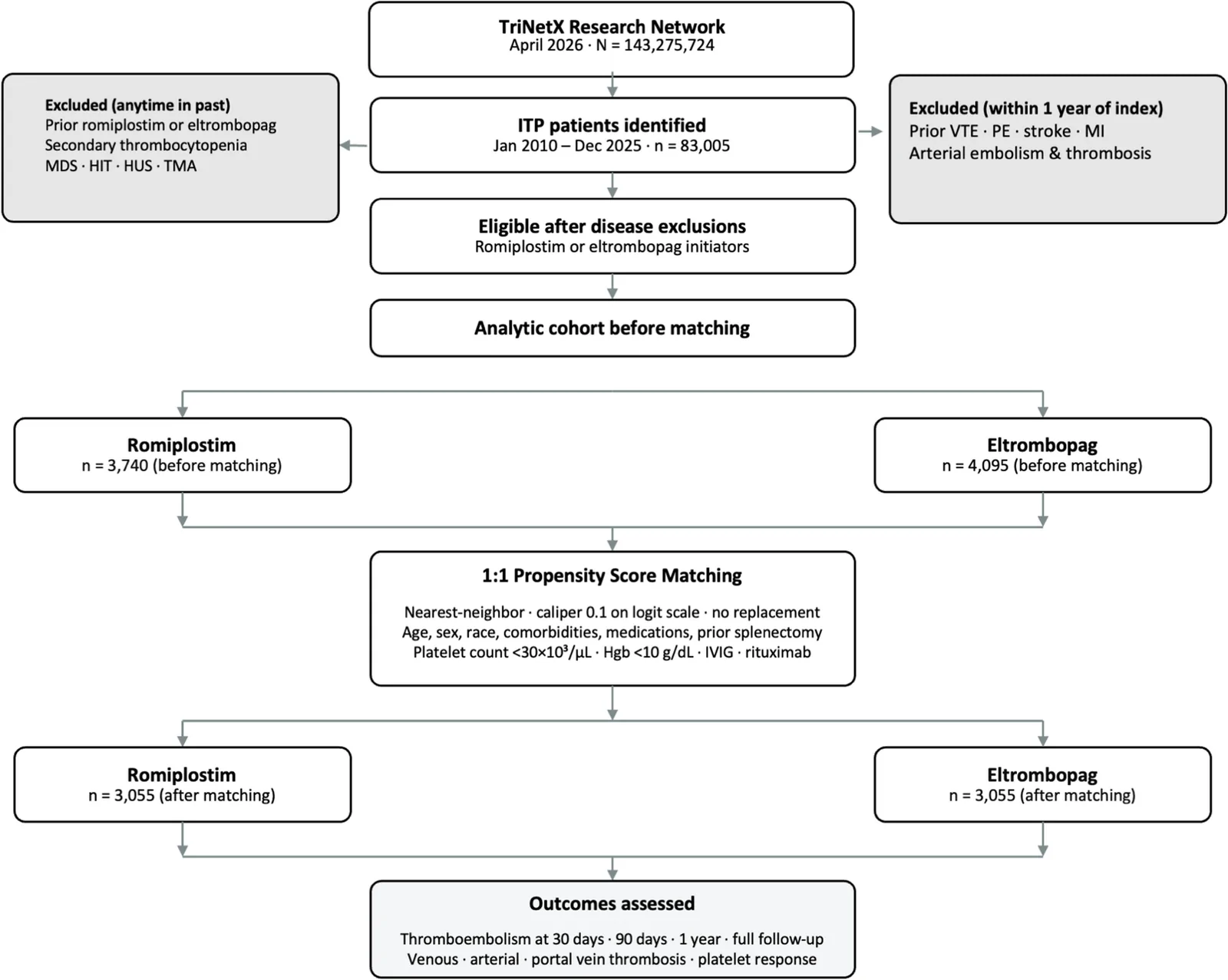
CONSORT-style cohort flow diagram

A total of 3,740 patients initiated romiplostim and 4,095 initiated eltrombopag before matching. Before matching, romiplostim-treated patients were older and had a higher prevalence of several cardiovascular and metabolic comorbidities, including hypertension, atrial fibrillation/flutter, type 2 diabetes mellitus, chronic kidney disease, and neoplasms. They were also more likely to have received corticosteroids, intravenous immunoglobulin, rituximab, and aspirin. Romiplostim users were also more likely to have severe thrombocytopenia (platelet count 0–30 × 10³/µL: 64.1% vs. 52.1%) and anemia (hemoglobin 10 g/dL: 36.1% vs. 22.2%), and had higher baseline AST levels. After application of exclusion criteria and 1:1 propensity score matching, 3,055 patients remained in each group (Fig. 1). Patients who could not be matched within the prespecified propensity score caliper were not included in the final matched analytic cohort. Most baseline characteristics were well balanced after matching, although residual imbalance persisted for hemoglobin < 10 g/dL (32.0% vs. 25.7%; standardized difference 0.141) (Table 1). Median follow-up was 971 days in the romiplostim group and 1,015 days in the eltrombopag group.

**Table 1:** Baseline characteristics before and after propensity score matching

Characteristic	Before Matching			After Matching		
	Romiplostim (*n* = 3,740)	Eltrombopag (*n* = 4,095)	SMD	Romiplostim (*n* = 3,055)	Eltrombopag (*n* = 3,055)	SMD
Demographics						
Age at index, mean ± SD	57.6 ± 17.8	54.3 ± 19.4	0.178	56.7 ± 18.0	56.9 ± 18.7	0.014
White, n (%)	2,591 (69.3%)	2,705 (66.1%)	0.069	2,095 (68.6%)	2,137 (70.0%)	0.030
Female, n (%)	1,903 (50.9%)	2,273 (55.5%)	0.093	1,620 (53.0%)	1,603 (52.5%)	0.011
Not Hispanic or Latino, n (%)	2,800 (74.9%)	2,905 (70.9%)	0.088	2,229 (73.0%)	2,261 (74.0%)	0.024
Black or African American, n (%)	455 (12.2%)	418 (10.2%)	0.062	347 (11.4%)	339 (11.1%)	0.008
Asian, n (%)	197 (5.3%)	299 (7.3%)	0.084	176 (5.8%)	163 (5.3%)	0.019
Comorbidities						
Essential hypertension, n (%)	1,547 (41.4%)	1,269 (31.0%)	0.217	1,143 (37.4%)	1,133 (37.1%)	0.007
Atrial fibrillation/flutter, n (%)	422 (11.3%)	286 (7.0%)	0.150	273 (8.9%)	270 (8.8%)	0.003
Type 2 diabetes mellitus, n (%)	808 (21.6%)	630 (15.4%)	0.161	580 (19.0%)	566 (18.5%)	0.012
Neoplasms, n (%)	1,283 (34.3%)	1,029 (25.1%)	0.202	943 (30.9%)	924 (30.2%)	0.014
Chronic kidney disease, n (%)	531 (14.2%)	358 (8.7%)	0.172	364 (11.9%)	340 (11.1%)	0.025
Obesity, n (%)	272 (7.3%)	223 (5.4%)	0.075	188 (6.2%)	203 (6.6%)	0.020
Systemic lupus erythematosus, n (%)	199 (5.3%)	158 (3.9%)	0.070	137 (4.5%)	140 (4.6%)	0.005
Tobacco use, n (%)	83 (2.2%)	49 (1.2%)	0.079	46 (1.5%)	48 (1.6%)	0.005
Procedure						
Splenectomy, n (%)	27 (0.7%)	30 (0.7%)	0.001	24 (0.8%)	22 (0.7%)	0.008
Medications						
Corticosteroids, n (%)	2,740 (73.3%)	2,699 (65.9%)	0.160	2,125 (69.6%)	2,105 (68.9%)	0.014
IVIG, n (%)	1,477 (39.5%)	954 (23.3%)	0.354	939 (30.7%)	925 (30.3%)	0.010
Rituximab, n (%)	519 (13.9%)	434 (10.6%)	0.100	389 (12.7%)	381 (12.5%)	0.008
Apixaban, n (%)	153 (4.1%)	149 (3.6%)	0.023	119 (3.9%)	113 (3.7%)	0.010
Rivaroxaban, n (%)	51 (1.4%)	53 (1.3%)	0.006	41 (1.3%)	42 (1.4%)	0.003
Warfarin, n (%)	106 (2.8%)	90 (2.2%)	0.041	74 (2.4%)	75 (2.5%)	0.002
Aspirin, n (%)	412 (11.0%)	324 (7.9%)	0.106	298 (9.8%)	284 (9.3%)	0.016
Clopidogrel, n (%)	77 (2.1%)	69 (1.7%)	0.028	61 (2.0%)	47 (1.5%)	0.035
Atorvastatin, n (%)	578 (15.5%)	525 (12.8%)	0.076	443 (14.5%)	435 (14.2%)	0.007
Systemic contraceptives, n (%)	100 (2.7%)	162 (4.0%)	0.072	86 (2.8%)	91 (3.0%)	0.010
Laboratory Values						
Platelet count 0–30 × 10³/µL, n (%)	2,399 (64.1%)	2,133 (52.1%)	0.246	1,801 (59.0%)	1,804 (59.1%)	0.002
Hemoglobin < 10 g/dL, n (%)	1,350 (36.1%)	908 (22.2%)	0.310	978 (32.0%)	784 (25.7%)	0.141
ALT, mean ± SD (U/L)	37.7 ± 66.7	31.4 ± 70.1	0.093	36.0 ± 56.7	32.5 ± 77.9	0.051
AST, mean ± SD (U/L)	35.3 ± 52.5	28.1 ± 36.4	0.159	33.6 ± 51.1	29.2 ± 40.2	0.095

Abbreviations: *IVIG* intravenous immunoglobulin, *ALT* alanine aminotransferase, *AST* aspartate aminotransferase, *SD* standard deviation, *SMD* standardized mean difference. Standardized differences < 0.10 indicate adequate balance after matching

Race and ethnicity categories reflect TriNetX coding and are not mutually exclusive

Romiplostim was associated with a higher cumulative risk of any thromboembolism than eltrombopag across all evaluated follow-up intervals: 30 days (7.2% vs. 5.7%; RR 1.27, 95% CI 1.05–1.54; *p* = 0.014), 90 days (10.7% vs. 7.9%; RR 1.36, 95% CI 1.16–1.59; *p* < 0.001), 1 year (14.9% vs. 12.2%; RR 1.22, 95% CI 1.07–1.39; *p* = 0.002), and full follow-up (22.5% vs. 19.2%; RR 1.17, 95% CI 1.06–1.29; *p* = 0.002) (Table 2).

**Table 2:** Thrombotic outcomes and platelet response after propensity score matching: Romiplostim vs. Eltrombopag in ITP

Outcome	Romiplostim (*n* = 3,055) n (%)	Eltrombopag (*n* = 3,055) n (%)	Risk Ratio (95% CI)	*p*-value
30-Day Outcomes				
Any thromboembolism	221 (7.2%)	174 (5.7%)	1.27 (1.05–1.54)	0.014
Venous thromboembolism	150 (4.9%)	113 (3.7%)	1.33 (1.05–1.69)	0.020
Pulmonary embolism	59 (1.9%)	44 (1.4%)	1.34 (0.91–1.98)	0.136
Deep vein thrombosis	61 (2.0%)	57 (1.9%)	1.07 (0.75–1.53)	0.710
Arterial thromboembolism	86 (2.8%)	72 (2.4%)	1.19 (0.88–1.63)	0.259
Acute myocardial infarction	39 (1.3%)	31 (1.0%)	1.26 (0.79–2.01)	0.336
Cerebral infarction	42 (1.4%)	38 (1.2%)	1.11 (0.72–1.71)	0.653
Platelet count, mean ± SD (×10³/µL)	141.8 ± 166.5	134.5 ± 156.8	-	0.114
90-Day Outcomes				
Any thromboembolism	328 (10.7%)	242 (7.9%)	1.36 (1.16–1.59)	< 0.001
Venous thromboembolism	214 (7.0%)	162 (5.3%)	1.32 (1.08–1.61)	0.006
Pulmonary embolism	86 (2.8%)	62 (2.0%)	1.39 (1.01–1.92)	0.046
Deep vein thrombosis	89 (2.9%)	84 (2.7%)	1.06 (0.79–1.42)	0.700
Arterial thromboembolism	145 (4.7%)	100 (3.3%)	1.45 (1.13–1.86)	0.003
Acute myocardial infarction	63 (2.1%)	44 (1.4%)	1.43 (0.98–2.10)	0.064
Cerebral infarction	70 (2.3%)	54 (1.8%)	1.30 (0.91–1.84)	0.147
Platelet count, mean ± SD (×10³/µL)	152.5 ± 169.6	138.6 ± 135.7	-	0.001
1-Year Outcomes				
Any thromboembolism	456 (14.9%)	374 (12.2%)	1.22 (1.07–1.39)	0.002
Venous thromboembolism	306 (10.0%)	247 (8.1%)	1.24 (1.06–1.45)	0.009
Pulmonary embolism	137 (4.5%)	98 (3.2%)	1.40 (1.08–1.80)	0.009
Deep vein thrombosis	139 (4.5%)	131 (4.3%)	1.06 (0.84–1.34)	0.618
Arterial thromboembolism	213 (7.0%)	162 (5.3%)	1.32 (1.08–1.60)	0.007
Acute myocardial infarction	105 (3.4%)	80 (2.6%)	1.31 (0.99–1.75)	0.062
Cerebral infarction	103 (3.4%)	81 (2.7%)	1.27 (0.96–1.69)	0.100
Portal vein thrombosis	44 (1.4%)	19 (0.6%)	2.32 (1.36–3.96)	0.002
Platelet count, mean ± SD (×10³/µL)	148.2 ± 144.1	145.1 ± 127.0	-	0.405
Full follow-up				
Any thromboembolism	686 (22.5%)	588 (19.2%)	1.17 (1.06–1.29)	0.002
Venous thromboembolism	440 (14.4%)	371 (12.1%)	1.19 (1.04–1.35)	0.009
Pulmonary embolism	200 (6.5%)	148 (4.8%)	1.35 (1.10–1.66)	0.004
Deep vein thrombosis	218 (7.1%)	208 (6.8%)	1.05 (0.87–1.26)	0.615
Arterial thromboembolism	381 (12.5%)	314 (10.3%)	1.21 (1.05–1.40)	0.007
Acute myocardial infarction	192 (6.3%)	155 (5.1%)	1.24 (1.01–1.52)	0.041
Cerebral infarction	195 (6.4%)	161 (5.3%)	1.21 (0.99–1.48)	0.063
Portal vein thrombosis	66 (2.2%)	35 (1.1%)	1.89 (1.26–2.83)	0.002
Platelet count, mean ± SD (×10³/µL)	155.9 ± 140.7	154.8 ± 125.7	—	0.749

Abbreviations: *CI* confidence interval, *RR* risk ratio, *SD* standard deviation. RR and 95% CI were not calculated for continuous platelet count outcomes (shown as -); p-values for platelet count represent independent samples t-test results. Portal vein thrombosis is reported only at 1 year and full follow-up because 30-day and 90-day event counts were below the TriNetX reporting threshold for small cell sizes

Venous thromboembolism was also consistently more frequent with romiplostim, with significant differences at 30 days (4.9% vs. 3.7%; RR 1.33; *p* = 0.020), 90 days (7.0% vs. 5.3%; RR 1.32; *p* = 0.006), 1 year (10.0% vs. 8.1%; RR 1.24; *p* = 0.009), and full follow-up (14.4% vs. 12.1%; RR 1.19; *p* = 0.009). Pulmonary embolism was significantly more common with romiplostim from 90 days onward, whereas deep vein thrombosis rates did not differ significantly between groups at any time point.

Arterial thromboembolism was higher with romiplostim at 90 days (4.7% vs. 3.3%; RR 1.45, 95% CI 1.13–1.86; *p* = 0.003), 1 year (7.0% vs. 5.3%; RR 1.32, 95% CI 1.08–1.60; *p* = 0.007), and full follow-up (12.5% vs. 10.3%; RR 1.21, 95% CI 1.05–1.40; *p* = 0.007), but not at 30 days. Acute myocardial infarction was significantly more frequent with romiplostim only over full follow-up (6.3% vs. 5.1%; RR 1.24, 95% CI 1.01–1.52; *p* = 0.041), whereas cerebral infarction did not differ significantly between groups. Portal vein thrombosis was uncommon but occurred more frequently with romiplostim at 1 year (1.4% vs. 0.6%; RR 2.32, 95% CI 1.36–3.96; *p* = 0.002) and over full follow-up (2.2% vs. 1.1%; RR 1.89, 95% CI 1.26–2.83; *p* = 0.002)(Table 2). Portal vein thrombosis was not reported at 30 or 90 days because event counts were below the TriNetX small-cell reporting threshold.

Mean platelet counts were similar between groups at 30 days, 1 year, and full follow-up. At 90 days, romiplostim was associated with a modestly higher mean platelet count than eltrombopag (152.5 ± 169.6 vs. 138.6 ± 135.7 × 10³/µL; *p* = 0.001), although both groups achieved platelet counts above clinically relevant treatment thresholds (Table 2).

##  Discussion

In this propensity score-matched retrospective cohort study of more than 6,000 adults with ITP, romiplostim was associated with higher cumulative thromboembolic risk than eltrombopag across prespecified follow-up windows. Differences were observed for both venous and arterial outcomes, while deep vein thrombosis and cerebral infarction did not differ significantly between groups. Mean platelet counts were broadly similar, suggesting that the observed thrombotic differences were not explained primarily by higher platelet counts.

These findings contrast with prior pharmacovigilance analyses that suggested higher thrombosis signals with eltrombopag rather than romiplostim [13]. However, such studies are inherently limited by reporting bias, lack of adjudicated outcomes, and inability to adjust adequately for confounding. Our findings suggest that, in a matched real-world ITP cohort, comparative thrombotic risk may differ from signals observed in spontaneous reporting databases.

The observation of higher cumulative thrombotic risk with romiplostim despite broadly similar platelet counts raises mechanistic questions, although this study was not designed to establish biological causality. Romiplostim binds the extracellular domain of the TPO receptor at the same site as endogenous thrombopoietin, whereas eltrombopag binds the transmembrane domain, activating distinct intracellular signaling cascades with differential AKT/ERK1/2 pathway engagement [18, 19]. TPO-RA treatment has been shown to enhance procoagulant activity through increased PAI-1 levels, greater phosphatidylserine exposure on apoptotic platelets, and elevated microvesicle-associated thrombin generation [20, 21]. However, none of these studies directly compared procoagulant effects between romiplostim and eltrombopag, leaving open whether differential signaling translates into measurably different prothrombotic profiles.

Portal vein thrombosis was uncommon but occurred more frequently among romiplostim-treated patients at 1 year and full follow-up. The romiplostim prescribing information includes a warning for portal vein thrombosis in patients with and without chronic liver disease, and case reports have described this complication with TPO-RA therapy [22, 23]. However, given the low absolute event rate and potential residual confounding from liver disease and other thrombotic risk factors, this finding should be interpreted cautiously and considered hypothesis-generating.

Several limitations should be acknowledged. As a retrospective EHR-based study, residual confounding from unmeasured or incompletely captured variables cannot be excluded, including ITP severity, disease duration, treatment selection, TPO-RA dosing, treatment duration, dose adjustments, and discontinuations. Because EHR coding may not fully distinguish primary ITP from autoimmune-associated secondary ITP, residual confounding from SLE, antiphospholipid syndrome, or other autoimmune thrombotic risk factors cannot be excluded. Although propensity score matching improved baseline balance, residual imbalance persisted for hemoglobin < 10 g/dl. Outcome ascertainment relied on ICD-10 coding and may be subject to misclassification. Outcomes were assessed cumulatively at prespecified follow-up windows rather than through time-to-event modeling; therefore, event timing, censoring, and differential duration of exposure could not be fully characterized.

In this large propensity score-matched real-world cohort of adults with ITP, romiplostim was associated with higher cumulative thromboembolic risk than eltrombopag across prespecified follow-up windows. Given the retrospective design, potential treatment-selection bias, and inability to fully characterize treatment exposure, these findings should not be interpreted as evidence of a causal treatment effect. Confirmation in prospective studies and real-world datasets with greater clinical granularity is warranted.

## Supplementary Information

Below is the link to the electronic supplementary material.

Supplementary Material 1 (DOCX 28.2 KB)

## Data Availability

The data that support the findings of this study were obtained from the TriNetX federated research network and are subject to platform restrictions; therefore, the raw data are not publicly available.
