# Supplementary material for: Comparative thromboembolic risk of romiplostim versus eltrombopag in immune thrombocytopenia: a propensity score-matched cohort study
Source: Ann Hematol. 2026 Jul 3;105(10):425. doi: 10.1007/s00277-026-07160-4 (PMC13597643; doi:10.1007/s00277-026-07160-4)
Supplement: Supplementary file 1 [file 277_2026_7160_MOESM1_ESM.docx]

**Supplementary Table S1. ICD-10 diagnosis codes used in the study**

This table summarizes the ICD-10 diagnosis codes used for cohort inclusion, exclusions, prior-event exclusions, outcomes, and diagnosis covariates used in propensity score matching. Medication definitions were based on RxNorm codes and laboratory variables on TriNetX/TNX identifiers; those non-ICD codes are not shown here.

| **ICD-10 code** | **Diagnosis / label in TriNetX report** | **Role in study** |
| --- | --- | --- |
| D69.3 | Immune thrombocytopenic purpura | Inclusion criterion for both cohorts |
| D61 | Other aplastic anemias and other bone marrow failure syndromes | Exclusion / alternative thrombocytopenia etiology |
| D69.5 | Secondary thrombocytopenia | Exclusion / alternative thrombocytopenia etiology |
| D46 | Myelodysplastic syndromes | Exclusion / alternative thrombocytopenia etiology |
| D75.82 | Heparin induced thrombocytopenia (HIT) | Exclusion / alternative thrombocytopenia etiology |
| D59.3 | Hemolytic-uremic syndrome | Exclusion / alternative thrombocytopenia etiology |
| M31.1 | Thrombotic microangiopathy | Exclusion / alternative thrombocytopenia etiology |
| I63 | Cerebral infarction | Prior thrombotic-event exclusion; study outcome; component of arterial thromboembolism and any thromboembolism |
| I21 | Acute myocardial infarction | Prior thrombotic-event exclusion; study outcome; component of arterial thromboembolism and any thromboembolism |
| I74 | Arterial embolism and thrombosis | Prior thrombotic-event exclusion; study outcome; component of arterial thromboembolism and any thromboembolism |
| I26 | Pulmonary embolism | Prior thrombotic-event exclusion; study outcome; component of venous thromboembolism and any thromboembolism |
| I82 | Other venous embolism and thrombosis | Prior thrombotic-event exclusion; study outcome; component of venous thromboembolism and any thromboembolism |
| I80 | Phlebitis and thrombophlebitis | Prior thrombotic-event exclusion |
| I81 | Portal vein thrombosis | Study outcome; component of venous thromboembolism and any thromboembolism |
| I82.4 | Acute embolism and thrombosis of deep veins of lower extremity | Deep vein thrombosis outcome |
| I10 | Essential (primary) hypertension | Propensity score matching covariate |
| I48 | Atrial fibrillation and flutter | Propensity score matching covariate |
| E11 | Type 2 diabetes mellitus | Propensity score matching covariate |
| C00-D49 | Neoplasms | Propensity score matching covariate |
| N18 | Chronic kidney disease (CKD) | Propensity score matching covariate |
| E66.0 | Obesity due to excess calories | Propensity score matching covariate |
| M32 | Systemic lupus erythematosus (SLE) | Propensity score matching covariate |
| Z72.0 | Tobacco use | Propensity score matching covariate |

Abbreviations: CKD, chronic kidney disease; HIT, heparin-induced thrombocytopenia; SLE, systemic lupus erythematosus.

Source: TriNetX Compare Outcomes Analysis report for the 30-day follow-up cohort comparison.
